# A Nanostructural Study of Mixed Carbon and Boron Nitride Nanotube Solutions in Superacid using Cryogenic Electron Microscopy

**DOI:** 10.1021/acs.langmuir.5c02961

**Published:** 2025-10-07

**Authors:** Asia Matatyaho Ya’akobi, Cedric J. S. Ginestra, Joe F. Khoury, Angel A. Martí, Matteo Pasquali, Yeshayahu Talmon

**Affiliations:** † Department of Chemical Engineering and the Russell Berrie Nanotechnology Institute (RBNI), 26747 Technion-Israel Institute of Technology, Haifa 3200003, Israel; ‡ Department of Chemical and Biomolecular Engineering, 3990 Rice University, 6100 Main Street, MS 369, Houston, Texas 77005, United States; § Department of BioEngineering, 3990 Rice University, 6100 Main Street, MS 369, Houston, Texas 77005, United States; ∥ Department of Chemistry, 3990 Rice University, 6100 Main Street, MS 369, Houston, Texas 77005, United States; ⊥ Department of Materials Science and NanoEngineering, 3990 Rice University, 6100 Main Street, MS 369, Houston, Texas 77005, United States; # The Smalley-Curl Institute, Rice University, 6100 Main Street, MS 369, Houston, Texas 77005, United States

## Abstract

We study the nanostructure of carbon nanotubes (CNTs) and boron nitride nanotubes (BNNTs) mixed in chlorosulfonic acid (CSA), a superacid that is a true solvent for both nanotube types. Cryogenic transmission electron microscopy (cryo-TEM) and cryogenic scanning electron microscopy (cryo-SEM) were used to analyze the phase behavior and morphological characteristics of the nanotube mixtures at various concentrations and proportions. We find that, surprisingly, CNTs and BNNTs mix well at all length scales. We image different phases, including isotropic and nematic liquid crystal, and find that the mixture composition influences the phase behavior and morphology. The consistent admixture of BNNTs and CNTs at small length scales observed in the isotropic and the nematic phases emphasizes the potential for developing hybrid materials that combine the unique properties of each nanotube type.

## Graphical abstract

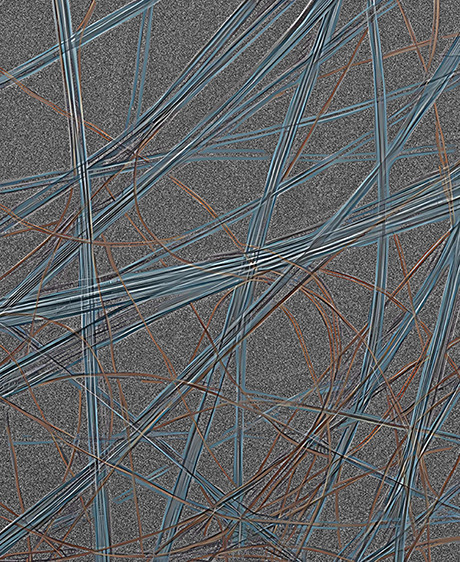

## Introduction

Carbon nanotubes (CNTs), first discovered by Iijima in 1991,^1^ can be described as seamless, one-dimensional cylindrical rolls of graphene sheets. Composed entirely of carbon, CNTs exhibit a remarkable morphology and atomic organization, leading to their chemical inertness.^2^ The sp^2^ bond within the graphitic structure of CNTs gives rise to their extraordinary molecular-level properties.^3,4^ At the nanoscale, CNTs possess exceptional mechanical strength and stiffness while maintaining a low density. Studies indicate that individual single-walled CNTs have an average Young’s modulus of ∼1 TPa and a tensile strength of ∼100 GPa.^5–7^ Additionally, individual CNTs exhibit impressive thermal conductivity, and depending on their atomic structure and diameter, single-walled CNTs can function as electron conductors or display semiconducting behavior.^8^

The discovery of CNTs spurred significant interest in analogous nanomaterials. Boron and nitrogen, positioned adjacent to carbon in the periodic table, form exceptionally strong sp^2^ bonds and were anticipated to form similar nanostructures to those of carbon. Boron nitride nanotubes (BNNTs) were first experimentally produced in 1995 by Chopra.^9^ Similarly to CNTs, BNNTs are seamless cylindrical hexagonal boron nitride (h-BN) sheet rolls. Individual BNNTs exhibit remarkable mechanical properties and low density, comparable to those of CNTs, while also demonstrating superior chemical stability and offering additional unique characteristics.^10–12^ For example, BNNTs can survive in air up to 900 °C, while CNTs are oxidized at ∼500 °C.^12^ Furthermore, BNNTs serve as electrical insulators, with a relatively uniform large bandgap regardless of their structure.^13,14^ Additionally, BNNTs possess radiation shielding capabilities due to their high neutron absorption cross-section.^15^

Given their complementary properties and similar structures, CNTs and BNNTs hybrid materials could be of great interest for various applications.^16^ The compatibility of CNTs and BNNTs as isoelectronic and isostructural nanomaterials suggests their potential combination in mixtures through van der Waals interactions. The fabrication of such hybrid materials would open new avenues for developing innovative materials with tailored functionality and synergistic properties derived from their multiple components, thus broadening their applicability. For example, incorporating BNNTs into CNT-based macroscopic assemblies could enhance their oxidation resistance and chemical stability, while also providing radiation shielding capabilities.^17^

The spontaneous molecular dissolution of CNTs and BNNTs in superacid through direct protonation^18^ has led to liquid-phase manufacturing processes to make multifunctional macroscopic structures that attain macroscopic ordering while maintaining the nanotubes’ intrinsic properties, such as length and sp^2^ hybridization.^19–21^ The thermodynamics of nanotube solutions can be approximately described by Onsager^22^ and Flory’s^23^ theories of polymeric and colloidal rigid-rod solutions.^24,25^ In solution, both CNTs and BNNTs form liquid crystals. As the concentration increases, the system changes from a dilute phase, where interactions between individual nanotubes are negligible, to a semidilute phase, where the rotation of the rods is inhibited, and then to an isotropic concentrated solution, where both rotational and translational movements are constrained.^24,26–29^ Due to entropic considerations and the restrictions on the degrees of freedom in the isotropic phase, there is a critical concentration at which the system separates into a liquid crystalline phase that coexists in equilibrium with the concentrated isotropic phase, referred to as the biphasic regime. The fraction of the ordered phase increases with concentration until a fully liquid crystalline phase is developed.^21,24,27,29,30^ This is a nematic liquid crystal, defined by a system where molecules are aligned along a preferred direction but lack positional order.^26–28^

The phase behavior of rod-like molecules in a solution is influenced by solvent strength^26,31^ and nanotube parameters, including length, diameter, polydispersity, and purity.^21,23,25,32,33^ In a mixed solution of CNTs and BNNTs, each nanotube type is anticipated to affect the phase behavior based on its structural properties. The primary differences between the CNTs and BNNTs in this study are their length, diameter, and number of walls (as detailed in the materials section), all of which impact their stiffness and persistence length. Specifically, longer, thinner nanotubes with fewer walls, as in the studied CNTs, are expected to exhibit semiflexible behavior with shorter persistence lengths, whereas shorter, thicker nanotubes with more walls, as with the studied BNNTs, behave as stiff, rigid rods with longer persistence lengths.^34,35^

According to classical polymer and colloid theories, nanotubes of different sizes and chemical characteristics are expected to demix thermodynamically.^36,37^ A substantial body of literature on rod-like colloid mixtures supports this, demonstrating that even small differences in particle dimensions or stiffness can significantly influence phase behavior. For example, Purdy et al.^38^ reported isotropic–nematic coexistence, and even nematic–nematic demixing at higher concentrations, in mixtures of thin and thick (PEG-grafted) rod-shaped viruses. Similarly, Puech et al.^39^ investigated a bidisperse rod system of short carbon nanotubes dispersed in a longer fd-virus nematic host. They found that the smaller, more flexible CNT “guest” rods aligned less strongly than the host viruses. These experimental findings agree with theoretical models that predict complex phase behavior in rod mixtures. Semenov and Subbotin,^40^ followed by Varga et al.,^41^ showed that bidisperse rod liquid dispersions can undergo isotropic–nematic separation and even nematic–nematic demixing, with phase coexistence regions often bounded by critical points. Altogether, this body of work indicates that small differences in rod length, diameter, or flexibility can drive phase segregation in otherwise homogeneous suspensions. Nevertheless, recent findings by Siqueira et al. indicate that solutions of CNTs with mixed origins and different properties form a homogeneous solution in minutes or hours with no signs of phase separation.^42^

Although pure CNT fluid phases have been explored extensively, and there is an emerging body of literature on BNNT fluid phases, the mixed CNT-BNNT system is still unexplored. In this study, we used cryogenic transmission electron microscopy (cryo-TEM) and cryogenic scanning electron microscopy (cryo-SEM) to investigate the morphological characteristics and phase behavior of mixtures of BNNTs and CNTs in chlorosulfonic acid (CSA), a thermodynamic solvent for both types of nanotubes.^18,31,43^ The mixed nanotube solutions can serve as “dope” for the liquid-phase processing of macroscopic multifunctional fibers and films, as previously reported with CNTs/CSA and BNNTs/CSA solutions.^19–21^ Here, we demonstrated the consistent admixture of CNTs and BNNTs on a small length scale in both the isotropic and nematic phases. The observations in this study lay the basis for the future development of hybrid materials through liquid-phase processing.

## Experimental Section

### Materials

The CNTs were obtained from Meijo Nano Carbon Co. Ltd. (Nagoya, Japan). The MEIJO EC1.5P CNTs used here are predominantly single-walled nanotubes with diameters ranging from 1 to 2 nm and lengths of several microns. The measured aspect ratio for these CNTs is ∼6400.^25^ The BNNTs were obtained from BNNT LLC (Newport News, VA). The SP10RX BNNTs used here primarily consist of double- and few-walled nanotubes, with an average diameter of approximately 5 nm and lengths of 1 to 2 μm. The measured aspect ratio for these BNNTs is ∼270. Chlorosulfonic acid (CSA), 99% pure, was purchased from Sigma-Aldrich.

### Preparation of CNT and BNNT Solutions in CSA

Before dissolution in superacid, CNTs and BNNTs were dried in an oven at 100 °C for a minimum of 1 h to eliminate any trapped moisture. The dissolution process of the dried materials took place in a glovebox, continuously purged with dry nitrogen gas. Low-concentration mixtures (below 0.4 wt %) were stirred overnight with a magnetic stirrer to achieve a uniform solution. In contrast, high-concentration solutions were mixed for 2 h at 3000 rpm using a DAC 150 SpeedMixer (Hauschild Eng., Germany).

### Cryo-TEM

We used thermal fixation to lower the vapor pressure of the system, preventing evaporation in the high-vacuum microscope, and to arrest the motion of molecules in the liquid.^44^ Specimens for cryo-TEM were prepared in a controlled environment vitrification system (CEVS)^45^ that is continuously purged with dry nitrogen gas to prevent reaction between CSA and moisture. A small droplet of the solution was applied on a perforated carbon film, supported on a 3 mm copper TEM grid. The droplet was blotted with a glass filter paper to form a thin film (<300 nm) and was then vitrified by plunging it into liquid nitrogen.

We perform cryo-TEM of low-concentration acidic solutions by a Thermo Fisher Talos 200C high-resolution TEM equipped with a field emission gun (FEG) and operated at an accelerating voltage of 200 kV.Cryo-specimens were maintained below a temperature of −175 °C within the microscope using a Gatan 626 cryo-holder, and imaged in the low-dose mode to reduce electron-beam radiation damage. Images were acquired digitally by an FEI Falcon III direct-imaging camera and the TIA software, utilizing “Volta phase plates” (FEI) to enhance image contrast.^47^

### Cryo-SEM

We also used the thermal fixation method to prepare cryo-SEM specimens, specifically for imaging high-concentration acid–based solutions that cannot be sufficiently thinned for cryo-TEM analysis. This approach allows cryo-SEM to complement cryo-TEM, enabling the study of the solutions across a broader range of concentrations. Specimens for cryo-SEM were prepared in the CEVS^45^ under a continuous flow of dry nitrogen gas to prevent superacid reaction with moisture. A 3 mm grid was dipped in the examined solution and placed between two gold planchettes. The specimen was then plunged into liquid nitrogen using specially designed tweezers,^48^ and freeze-fractured either with the BAF060 unit (Leica AG. Liechtenstein) at −167 °C or with the newer Leica ACE600 freeze-fracture system at −180 °C.

We used a FEG-equipped Zeiss Ultra Plus HR-SEM with a Leica VT100 cryo-system. Specimens were maintained below a temperature of −145 °C inside the microscope, and imaged without a conductive coating at low acceleration voltages, from 0.6 to 1.2 kV, to ensure electrical neutrality, thereby preventing charging and minimizing imaging artifacts. Micrographs were captured at a short working distance of 3–4.5 mm, using the in-the-column high-resolution secondary electron detector (the “InLens” detector in Zeiss terminology).

## Results and Discussion

The liquid phases of the pure materials used in this study, in CSA, have been previously investigated. For these CNTs, the isotropic-to-nematic phase transition was reported by Tsentalovich et al. at a concentration of 55 ppmw.^25^ In this work, CNT and BNNT concentrations are expressed on a weight basis (ppmw). While rigid rod phase behavior is more rigorously described using a volume basis (ppmv), use of a mass basis is more experimentally expedient, because the CNT or BNNT density must be determined through experimental measurement of the average aspect ratio and number of walls for the nanotube batch under study.^25,49^ Here, we choose to express solution concentrations on a weight basis, knowing that this value is similar to the volume basis concentration (because ρ_CNT_ ≈ ρ_CSA_ ≈ ρ_BNNT_), and this difference does not affect the analysis of these results. For BNNTs of aspect ratio ∼300 and diameter of 5.8 nm, Ginestra et al. documented the isotropic-to-nematic phase transition at a concentration of 170 ppmw,^21^ an order of magnitude lower than for CNTs with a similar aspect ratio. This difference is attributed to the residual attraction between adjacent BNNTs, which results from less effective charge stabilization relative to CNTs. It is important to note that earlier direct imaging studies on single-component systems consistently showed that, within the biphasic regime, macroscopic phase separation is rarely observed on short experimental time scales (hours to days), and the two phases typically coexist at the microscopic scale forming either tactoids of ∼10 to 100 μm size or “striated” structures where the liquid crystalline phase forms thin, elongated, seemingly endless structures (particularly for rods of thinner diameter, below ∼1 nm, and higher lengths, above ∼2 to 5 μm); at higher rod concentration, polydomain structures are always observed.^25,27,30,50–52^

### Cryogenic Transmission Electron Microscopy (cryo-TEM)

Cryo-TEM is a powerful technique for studying complex liquids in their native state. However, applying this method to systems with such a reactive solvent requires special methodologies.^53^ We employed our dedicated cryo-TEM methodology to investigate CNT/BNNT mixtures at low concentrations in CSA (up to 4000 ppmw). Above this concentration, the viscosity of the solution increases, and the specimen cannot be thinned adequately for TEM observation.

Figure 1 presents cryo-TEM micrographs of a BNNT and CNT mixture in CSA at a total concentration of 1000 ppmw and a weight ratio of 1:1 (500 ppmw CNTs and 500 ppmw BNNTs). In this concentration range, we find coexistence of isotropic and aligned phases, consistent with the isotropic cloud points of the neat CNT and BNNT solutions (55 ppm^25^ and 170 ppmw,^21^ respectively). Figure 1A shows an example of the isotropic phase of the solution, where thick and rigid nanotubes appear as cylinders (white arrows), while thinner and more flexible nanotubes appear as threads (black arrows). That allows us to distinguish between CNTs and BNNTs in the Cryo-TEM images. Specifically, CNTs exhibit a lengthened and more flexible morphology, with a small diameter of 1.5–2 nm, corresponding to the thread-like nanotubes. In contrast, BNNTs are characterized by higher rigidity and a larger 4–5 nm diameter, corresponding to the observed cylindrical nanotubes. The white arrowhead points to nontubular h-BN contamination originating from and commonly observed in BNNTs synthesized using the high-temperature–pressure method.^30^ Figure 1B illustrates an early stage of BNNT alignment into ordered bundles (white arrows). In contrast, Figure 1C displays a wider BNNT ordered domain (white arrows), coexisting with isotropically dispersed, individual CNTs within a concentrated phase, indicating that the solution is in the biphasic regime. These observations demonstrate the nanoscale mixing of CNTs and BNNTs, except in the nascent liquid crystalline phase of BNNTs shown in Figure 1B.

**Figure 1:**
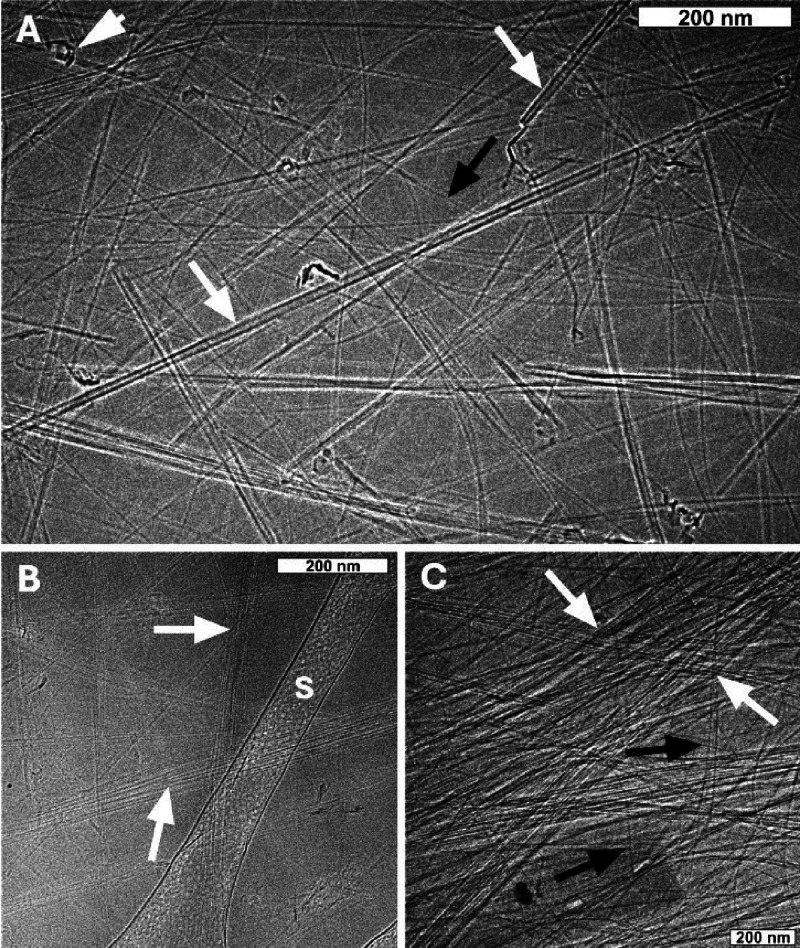
Mixture of 500 ppmw BNNTs and 500 ppmw CNTs in CSA (total nanotube concentration 1000 ppmw). (A) Sparse isotropic phase of combined rigid thick BNNTs (white arrow) and more flexible thin CNTs (black arrow). The white arrowhead points to the common nontubular h-BN contamination of HTP BNNTs.^30^ (B) Early-stage formation of BNNT liquid crystal, the white arrows point to BNNTs aligned in bundles. S denotes the perforated carbon support film on the TEM grid. (C) Concentrated nanotube phase of aligned BNNTs (white arrows) in equilibrium with isotropic CNTs (black arrows).

Figure 2 shows cryo-TEM images of a BNNT and CNT mixture in CSA at a total concentration of 1000 ppmw and a weight ratio of 1:3. To observe the alignment of the CNTs into the ordered phase, we increase the weight ratio of CNTs to BNNTs. Some CNTs are arranged in a sparse phase with aligned orientation (black arrows), coexisting with curved CNTs (black arrowheads) and dense bundles of BNNTs (white arrow). The relatively low degree of CNT alignment at this higher CNT to BNNT weight ratio, despite the significantly greater CNT aspect ratio, is consistent with the previously observed earlier alignment of BNNTs compared to CNTs.^21,25^ Note the tight curved CNT (black arrowhead) with an estimated radius of curvature on the order of 100 nm, which is unexpected given the large persistence length of CNTs (tens of microns).^34,54^ Such bent configurations are unlikely to arise from thermal fluctuations alone, and are probably a result of flow fields induced by shear forces applied during specimen preparation.

**Figure 2:**
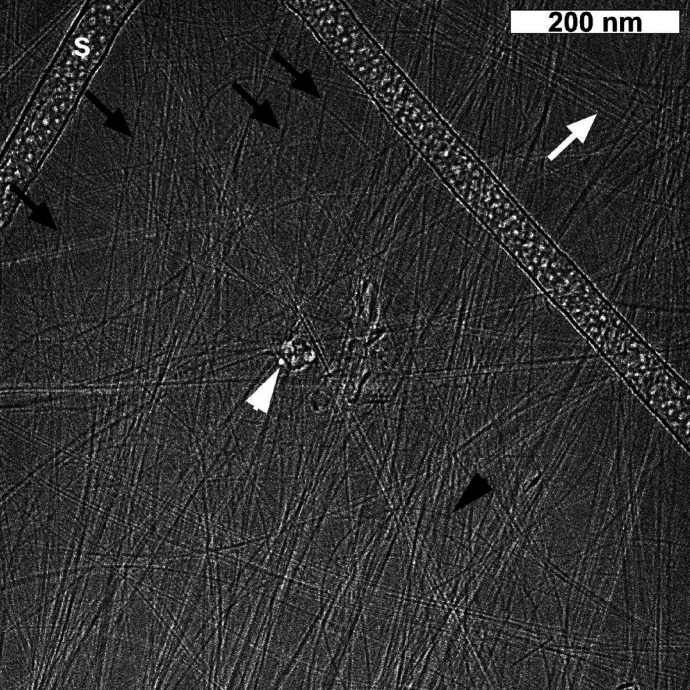
Mixture of 250 ppmw BNNTs and 750 ppmw CNTs in CSA (total nanotube concentration 1000 ppmw). At a CNT to BNNT weight ratio of 3:1, CNTs start to show loose alignment. Black arrows point to straight CNTs that coexist with more flexible CNTs (black arrowhead) and BNNT bundles (white arrow). A white arrowhead points to an h-BN particle. “S” denotes the perforated carbon support film on the TEM grid.

Figure 3 presents cryo-TEM images of a BNNT and CNT mixture in CSA at a total concentration of 4000 ppmw and a weight ratio of 1:1. This concentration shows an unusual morphology (Figure 3A): the BNNTs primarily appear aligned in bundles (white arrows), while the CNTs are distributed isotropically (black arrows), and display significant curvature. This unusual phase coexists with denser liquid crystalline domains, several micrometers wide, observed in other regions of the specimen (Figure 3B). The specific type of nanotube within the denser, ordered phase cannot be identified. However, this nematic phase is in equilibrium with a dilute isotropic phase of curved CNTs (black arrows) and BNNT bundles (white arrows).

**Figure 3:**
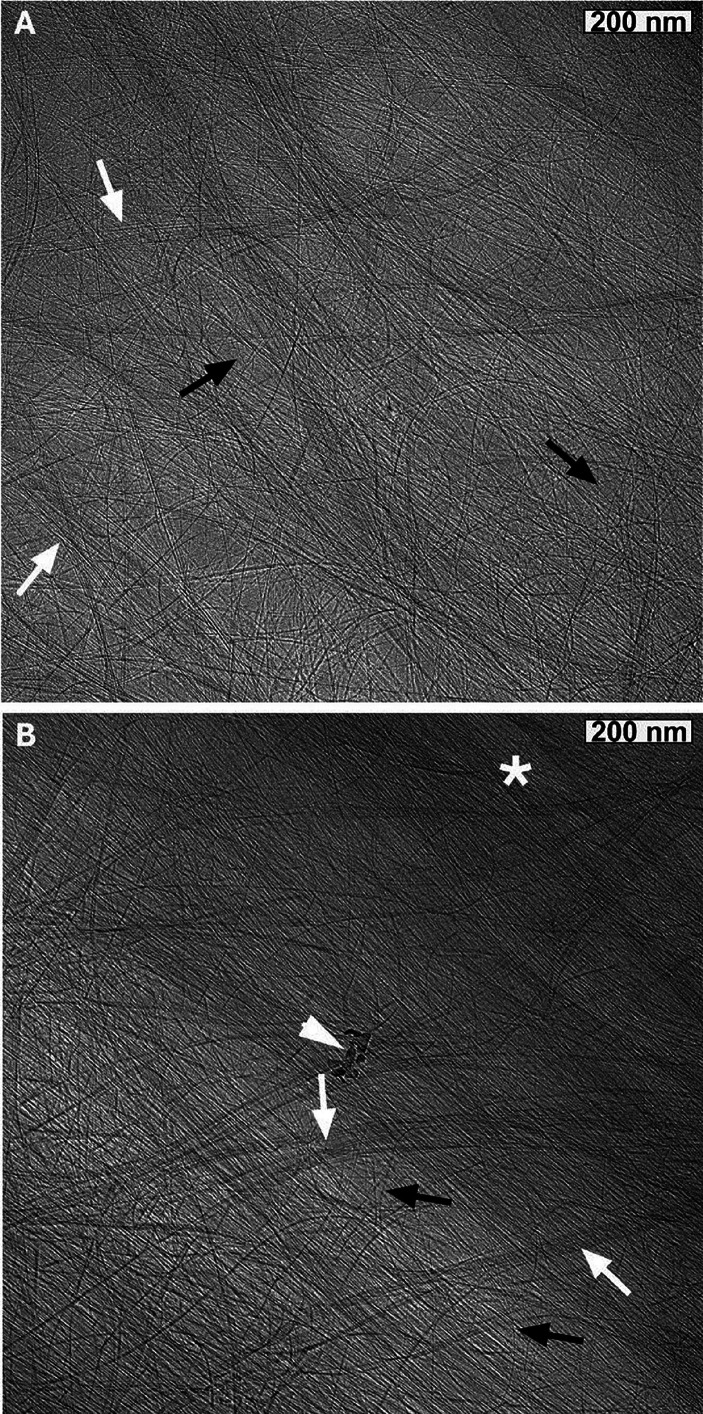
Mixture of 2000 ppmw BNNTs and 2000 ppmw CNTs in CSA (total nanotube concentration 4000 ppmw). (A) Crowded isotropic phase of BNNTs aligned in bundles (white arrows) and isotropic domains of CNTs (black arrows). (B) Highly concentrated, wide nematic liquid crystalline phase (denoted by “*”) with a superposed sparse isotropic phase. In the sparse isotropic region, individually randomly oriented CNTs (black arrows) and BNNT bundles (white arrows) are recognized, while in the dense liquid crystal phase, the specific type of nanotube cannot be identified. White arrowhead points to contamination.

Our cryo-TEM observations demonstrate the earlier arrangement of BNNTs into ordered domains at lower concentrations, as observed in a previous study.^21^ This early alignment of BNNTs can be attributed to their inherent rigidity and the possible residual attractive interaction between adjacent nanotubes. Furthermore, the cryo-TEM results clearly illustrate the admixture of BNNTs and CNTs within the dilute isotropic phases.

Figure 4 shows a nanotube loop, a phenomenon we observed in our studies only in the CNT and BNNT mixtures in CSA. It is worth mentioning that through their preparation, the solutions were mixed solely using a magnetic stir bar, without the application of sonication. The measured nanotube diameter is 5 nm, while the calculated nanotube length is approximately 0.9 μm, determined from the measured ring diameter. These dimensions, which correspond to the case of a closed nanotube ring, fit the characteristics of the studied BNNTs. However, assuming a closed loop with a radius of ∼150 nm implies a very high bending energy, 10^7^ times the thermal energy, due to the high persistence length of BNNTs (∼7 mm).^35,55^ Moreover, if the nanotube ends were connected via a polar bond, the resulting structure would likely exhibit a wedge-shaped junction rather than a perfectly circular ring. Therefore, it is more likely that the observed loop represents a single CNT looped around itself,^56,57^ or being bent by external forces.

**Figure 4:**
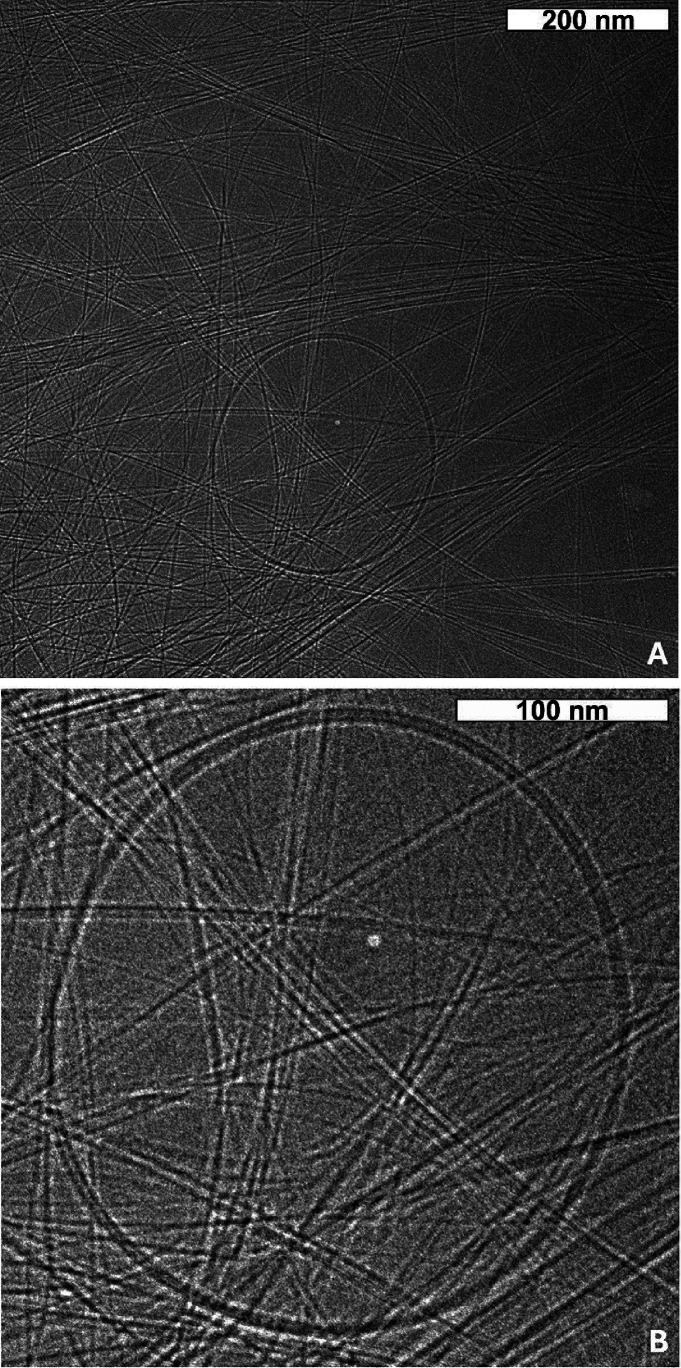
Closed nanotube in a mixture of 2000 ppmw BNNTs and 2000 ppmw CNTs in CSA. (A) Nanotube loop, which was observed only in mixed nanotube solutions. (B) Higher magnification of the nanotube ring. Note the higher presence of thin CNTs in this area.

By studying the mixed solution of BNNTs and CNTs using cryo-TEM, we were able to compare the electron beam radiation damage sustained by each type of nanotube. Figure 5 shows images of the same specimen area, showing both CNTs and BNNTs following increasing electron exposures. Figure 5A presents the initial image of the series, acquired with an electron exposure of 9 e^–^/Å^2^. At this low electron dose, the nanotubes are barely visible. However, further exposure of 17 e^–^/Å^2^ during the acquisition of the second image (for a cumulative exposure of 26 e^–^/Å^2^, Figure 5B) enhances the contrast of the nanotubes due to preferential radiation damage occurring at the nanotube/acid interface.^30,46^ The nanotube contrast is enhanced by a white halo around the nanotubes, a common feature used to visualize nanotubes in cryo-TEM imaging.^30,46^ At this stage, most radiation damage is observed on the carbon support film, with initial bubble formation beginning at the BNNT/CSA interface (white arrows). After a total exposure of 44 e^–^/Å^2^ (Figure 5C), radiation damage extends along the already-damaged BNNTs (white arrowheads), while CNTs remain largely undamaged. Severe radiation damage is observed on the BNNTs and the carbon support film after a total electron exposure of 70 e^–^/Å^2^ (Figure 5D), whereas the CNTs exhibit resistance to the electron beam. Our previous studies on BNNTs indicated that radiation damage tends to begin on nanotube-defected sites; therefore, the higher defect density in BNNTs relative to CNTs accounts for the predominance of radiation damage observed in BNNTs.^30^

**Figure 5:**
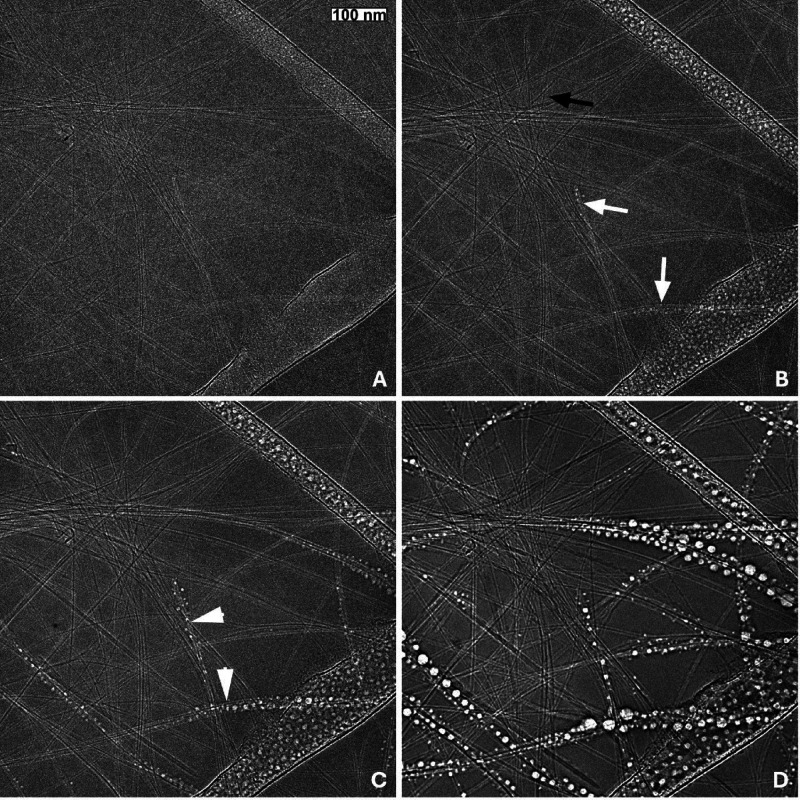
Radiation damage in cryo-TEM of a BNNT and CNT mixture in CSA. A series of the same specimen area, including CNTs and BNNTs, with an increasing electron dose of (A) 9 e^–^/Å^2^, (B) 26 e^–^/Å^2^, (C) 44 e^–^/Å^2^, and (D) 70 e^–^/Å^2^. The nanotubes are barely visible at the lowest electron dose (A). As the electron dose increases, the radiation damage preferentially occurs on the nanotube/CSA interface, enhancing their contrast (B). Excessive radiation damage is seen as bubbles on the BNNTs (white arrow), while the CNTs and the CSA far from the BNNTs remain largely unaffected (black arrow). Further exposure spreads radiation damage (white arrowheads) along the already damaged BNNTs (C). Despite the noticeable radiation damage observed on BNNTs, the CNTs exhibit high durability relative to the electron beam (D).

Radiation damage often seen as bubbles, actually small holes, is frequently observed in cryo-TEM of aqueous specimens,^58,59^ typically attributed to water radiolysis, the formation of free-radicals, and their attack on organic materials in the vicinity. A similar mechanism occurs in this case, where the CSA undergoes electron-beam-induced radiolysis that appears at the nanotube/superacid interface.^30,46,53^ we assume that the interface exhibits increased reactivity due to the delocalized positive charge on the nanotubes, resulting from their protonation in the superacid.

### Cryogenic Scanning Electron Microscopy (cryo-SEM)

The specialized cryo-SEM methodology that we developed for highly reactive CSA solutions enables us to extend our investigation to high-concentration solutions that cannot be imaged using cryo-TEM.^53^ Because CNTs and BNNTs are composed of light elements, we do not expect to observe atomic number contrast between them. Instead, as demonstrated in a previous study,^60^ we can obtain micrograph contrast between CNTs and BNNTs using the in-the-column high-resolution secondary electron detector. This contrast arises from differences in the electron conductivity of the nanotubes, resulting in charge accumulation on the nanotubes during exposure to the electron beam, producing voltage contrast. When the specimen is exposed to the electron beam for enough time to induce surface charging, electrons accumulate on the insulating BNNTs, resulting in their bright appearance. In contrast, the conductive CNTs become positively charged, making them appear darker. It is worth noting that initial exposure of the imaged area to the electron beam etches the acid from the surface, thereby enhancing nanotube visibility.^53,60^ With continued exposure, voltage contrast gradually develops, as described above.

In Figure 6 we show cryo-SEM micrographs of a BNNT and CNT mixture in CSA at a total concentration of 10,000 ppmw and a weight ratio of 3:1. At this concentration and BNNT:CNT weight ratio, the solution shows the coexistence of an isotropic phase (Figure 6A) and an ordered nematic phase (Figure 6B), indicating that the solution is in the biphasic regime. In both the concentrated isotropic and the ordered phases, bright-appearing BNNTs (white arrows) are observed alongside dark-appearing CNTs (black arrows), demonstrating their admixture within each phase. In the nematic liquid crystalline phase, a general alignment of the nanotubes into a preferred direction is observed, however, it shows a low degree of alignment and relatively disorganized packing, probably due to the higher concentration of the shorter BNNTs in the mixture.

**Figure 6:**
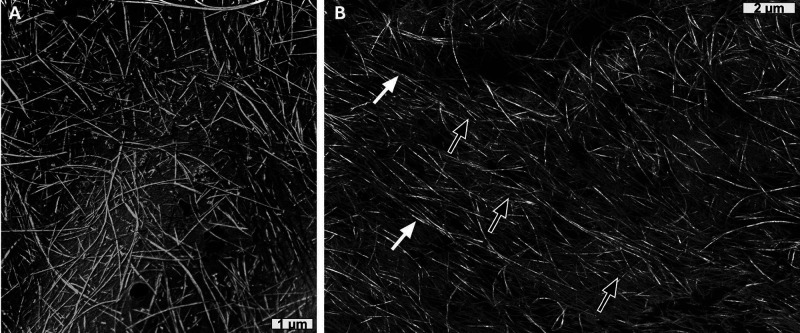
Mixture of 7500 ppmw BNNTs and 2500 ppmw CNTs in CSA (total nanotube concentration 10,000 ppmw). (A) Concentrated isotropic phase clearly shows the coexistence of bright-appearing BNNTs and dark-appearing CNTs within the phase. (B) Ordered phase in which the bright-appearing BNNTs (white arrows) are observed to be aligned in the same direction alongside the dark-appearing CNTs (black arrows). Although there is a general alignment of the nanotubes in this phase toward a predominant direction, they do not display a high level of alignment or dense packing.

Figure 7 presents cryo-SEM micrographs of a BNNT and CNT mixture in CSA at a total concentration of 10,000 ppmw and a weight ratio of 1:1. In contrast to the mixture shown in Figure 6, which has the same total concentration but a different BNNT:CNT weight ratio, this 1:1 solution predominantly shows ordered nematic phases in equilibrium with small isotropic domains. This observation indicates a biphasic solution approaching the formation of a fully liquid crystalline solution, showing how the nanotube composition affects the solution phase behavior. The higher proportion of longer CNTs in this mixture, compared to the more polydisperse BNNTs, reduces the overall polydispersity of the system, thereby promoting the development of a fully liquid crystalline solution.

**Figure 7:**
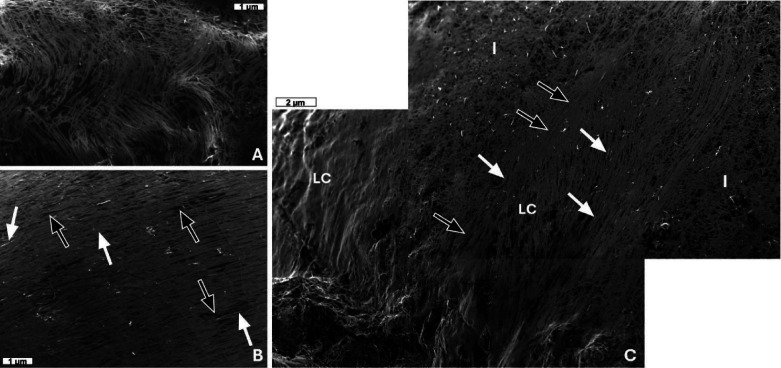
Mixture of 5000 ppmw BNNTs and 5000 ppmw CNTs in CSA (total nanotube concentration 10,000 ppmw). (A) Liquid crystalline phase made of several ordered nanotube domains, differently oriented and exhibiting a short length scale. (B) Highly ordered and densely packed liquid crystal, where bright-appearing BNNTs (white arrows) and dark-appearing CNTs (black arrows) are identifiable. (C) Photomontage of a large liquid crystalline domain (denoted by “LC”), which has a long length scale, likely due to the longer CNTs (black arrows). The short BNNTs are integrated into the CNT nematic phase (white arrows). Small disordered isotropic domains (denoted by “I”) are observed in equilibrium with the liquid crystalline phase, indicating a biphasic solution that is approaching the concentration at which a fully liquid crystalline solution is developed.

In Figure 7A, the liquid crystalline phase consists of several nanotube-ordered domains. Each domain is differently orientated and is characterized by a relatively short length scale, which may indicate that the shorter BNNTs primarily form these aligned domains. Another possibility is that this may be a region where the director is spatially changing, corresponding to the earlier light microscopy studies of CNT liquid crystals that show they do not have sharp domain boundaries, but rather, domains merge into each other via boundary regions where the director changes spatially. In contrast, in Figure 7B we observe a wide, highly ordered, and densely packed liquid crystal. Bright-appearing BNNTs (white arrows) and dark-appearing CNTs (black arrows) can be identified within this ordered nematic phase. However, the high degree of alignment in this phase is likely attributed to the predominant presence of longer CNTs. Figure 7C presents a photomontage of a large liquid crystal, characterized by a long length scale associated with the longer CNTs (black arrows). Bright-appearing BNNTs are observed interspersed among the CNTs within the ordered nematic phase. The small, disordered domains observed, denoted by I, could be a deformed director field pointing out of the image plane or a result of a thermal fluctuation.

Although the cryo-SEM micrographs in Figure 7 show distinct morphologies of the nematic phase, likely influenced by the predominant nanotube type within each phase, the findings throughout this research consistently demonstrate the admixture of BNNTs and CNTs at small length scales. The existence of domains that are more densely populated with one nanotube type within these solutions is expected and likely due to rigid rod phase behavior, rather than chemical incompatibility. Longer rods tend to assemble into nematic domains at lower concentrations than shorter rods, even in mixed solutions, and these results show that the shorter rods are not excluded from these assemblages. This admixture, which is surprising, given the predicted phase separation by classical polymer theories, is apparent within both the nematic and the isotropic phases, showing that the different types of nanotubes do not phase-separate in the mixed solutions.

## Conclusions

In this study, we investigated the nanostructure of CNT and BNNT mixtures in CSA, demonstrating the unique behavior and interactions of these two nanotube types when dissolved together in CSA, a superacid. We observed by cryo-TEM and cryo-SEM that the nanotube mixtures form admixed nematic domains that exhibit various morphological characteristics, influenced by their concentration and weight ratios in the solution.

Cryo-TEM studies showed that at lower concentrations, BNNTs tend to organize into ordered domains earlier than CNTs, despite their shorter length. This behavior is attributed to the BNNT rigidity and the residual interactions between adjacent nanotubes, which promote the earlier BNNT alignment.^21,43^ CNT alignment was observed in low-concentration solutions at a higher CNT:BNNT weight ratio. Cryo-SEM micrographs demonstrated the effect of the CNT:BNNT weight ratio on the morphology of the liquid crystalline phase at higher concentrations. Notably, the admixture of BNNTs and CNTs at a small length scale was consistently observed across all examined concentrations, both in the isotropic and the nematic phases, indicating the potential for developing hybrid materials that combine the unique properties of both nanotube types.

The results of this research contribute to the understanding of the nanoscale interactions in nanotube mixtures. This knowledge is essential for future studies aimed at producing macroscopic hybrid nanotube-based materials through liquid phase processing, including multifunctional fibers and films. Such advancements will open new opportunities for developing advanced composite materials, paving the way for innovative applications.
